# Difficulties experienced by young people with Tourette syndrome in secondary school: a mixed methods description of self, parent and staff perspectives

**DOI:** 10.1186/s12888-016-0717-9

**Published:** 2016-01-20

**Authors:** Ruth Wadman, Cris Glazebrook, Charlotte Beer, Georgina M. Jackson

**Affiliations:** School of Psychology, University of Nottingham, Nottingham, NG7 2RD UK; Division of Psychiatry and Applied Psychology, University of Nottingham, Nottingham, NG7 2TU UK

**Keywords:** Tourette syndrome, Adolescence, School, Qualitative methods, Mixed methods

## Abstract

**Background:**

Tourette syndrome (TS) is a neurodevelopmental disorder characterised by motor and vocal tics. These involuntary movements and vocalizations can have a negative impact in the school environment. The paper presents a mixed methods description of the difficulties experienced by UK students with TS in secondary school, drawing on multiple perspectives.

**Methods:**

Thirty-five young people with TS (11 to 18 years), their parents (*n* = 35) and key members of school staff (*n* = 54) took part in semi-structured interviews about TS-related difficulties in secondary school. Theme analysis was used to identify school difficulties reported by the young people, before moving on to analysis of the parents’ and staff members’ transcripts. The most frequently occurring themes from the young people’s accounts were then quantified in order to examine the level of agreement between informants and the association with clinical symptom severity.

**Results:**

A range of TS-related difficulties with academic work, and social and emotional well-being in school were reported by young people, parents and staff. Three superordinate themes are described: 1) TS makes school work more difficult, 2) Negative response to TS from staff and fellow students and 3) TS makes it more difficult to manage emotions in school. The three difficulties most frequently reported by the young people were problems concentrating in class, unhelpful responses by school staff to tics and difficulties with other students such as name-calling and mimicking tics. Additional difficulties reported by more than a quarter of young people related to homework, examinations, writing, anxiety and managing anger in school. Having more severe motor tics was associated with reporting difficulties with homework and handwriting, whereas having more severe phonic tics was associated with reporting unhelpful responses from staff. Young people and parents agreed more strongly with each other than they did with staff regarding school difficulties faced by individuals, and staff generally reported fewer TS-related difficulties.

**Conclusions:**

TS can present a barrier to learning in several ways and can also affect interactions with others and emotional experiences in secondary school. Implications for supporting secondary school-aged students with TS are considered.

## Background

As many as 1 in 100 school-aged children are thought to have Tourette syndrome (TS), a condition involving involuntary motor and phonic tics that have been present for more than 12 months [1]. Tics typically first appear between the ages of 4 and 6 years and reach peak severity between 11 and 14 years, then become less severe into adulthood [2]. TS does not affect intellectual ability per se but has a high prevalence rate with comorbid learning disabilities [3]. Furthermore, around 90 % of the TS population present with comorbid neuropsychiatric conditions or associated symptoms [4]. Associated conditions frequently co-occurring with TS include Attention Deficit Hyperactivity Disorder (ADHD) and Obsessive Compulsive Disorder (OCD). The majority of young people with TS are educated within mainstream schools, although a higher percentage of young people in special educational settings have tics or meet the diagnostic criteria for TS (compared to regular educational settings) [5, 6].

Tics fluctuate in type, severity and frequency and can be supressed (with effort and with varying success) for short periods of time [7, 8]. Thus, a student with TS will present a changeable profile with periods when their tics are more severe and disruptive in school. TS has the potential to impact significantly on school experience and educational attainment. A retrospective US clinical cohort study found 46 % of students with TS had school-related problems indicated by placement in special education, grade retention or learning disability [9]. Furthermore, a Swedish study of children with TS found that 79 % were considered by teachers to have significant academic and/or socioemotional adjustment problems in the classroom [10]. Students with TS and comorbid diagnoses may be at particular risk for school difficulties. For example, a Danish clinical cohort study found that 59 % of young people with TS had an educational difficulty requiring support but those with TS and a comorbid disorder were more likely to be in a special educational setting and to have changed schools at least once due to TS-related problems [11].

Schools can play an important role in promoting health and wellbeing, including mental health, and can limit the impact of risk factors such as neurodevelopmental disability for students [12, 13]. Recent European clinical guidelines emphasize the importance of educating schools about TS in addition to providing clinical behavioral interventions for young people with TS [14]. However, relatively little empirical work has examined the nature of the specific challenges encountered by students with TS in school.

An early survey of adults and children via a TS support organisation found the most commonly reported education problem (past or present) was concentration (82 %), followed by paying attention (78 %) and performance on time-limited tasks (71 %) [15]. In addition, 78 % of respondents rated school personnel as “not knowledgeable at all” about TS. In a US survey of parents, half reported that their child (aged between 6 and 17 years) had moderate or significant academic difficulties related to tics, in particular, difficulties with reading and handwritten work due to motor tics and an unwillingness to speak in class due to vocal tics [16]. Parents also reported that young people with TS could encounter tic-related problems with peers in school such as teasing (39 %) and peer rejection (28 %) [16]. More recently, a large survey of a US support organisation for TS found over a quarter of members (26 %) reported peer victimization [17]. Similar levels of peer victimization (27 %) were reported in a study of young people with TS compared to the 9 % reported by their peers without TS [18]. These studies, carried out in North America, suggest a considerable proportion of children and adolescents with TS experience academic difficulties in school as a result of TS as well as experiencing problems with their peers.

A recent focus group study examined the perceptions of Spanish adolescents with TS (11–18 years), their parents and health professionals about TS-related school, social and family problems [19]. Health professionals reported that TS primarily affected learning in school and social adjustment. Adolescents reported a number of difficulties in school including slower learning, attention problems and conflict with teachers. Parents reported that the lack of understanding of TS shown by teachers and peers led to difficulties in school. This suggests that schools may not be realising their potential to tackle problems caused by TS symptoms. No study has explored how the views and experiences of the young person with TS accord with those of their parents and teachers.

The educational experiences of UK students with TS have not been studied in depth and clinical opinion suggests that many young people with TS are not satisfactorily supported by education services [20]. A UK study found school children with TS, aged between 8 and 18, had poorer quality of life self-ratings in the school domain compared to typically developing children [21]. In addition, qualitative focus group interviews with young people with TS revealed two particular challenges in school; that the effort to control tics was distracting and being different from peers may make students a target for bullying [21].

### The present study

The present study used a case study approach to explore TS-related school difficulties experienced by young people with TS attending mainstream secondary school from the perspective of the young person, their parent/s and school staff members. Previous TS research in this area has tended to explore a single perspective (e.g., parent or young person). Using interviews and theme analysis with multiple informants offers the opportunity to explore different perspectives on the school experience, as well as identifying areas of possible discrepancy or conflict, and will thus provide insight into ways to improve the educational and psychosocial development of young people with TS within a school setting. Previous studies of TS-related school difficulties have included children across a wide age range and (with the exception of Rivera-Navarro et al. 2014) have not focused on the difficulties experienced by adolescents in secondary school. Secondary schools are likely to offer additional challenges such as greater academic pressures, subject specific teachers and larger year groups. This is the first study to explore TS-related difficulties in mainstream secondary schools in the UK. As part of our analysis we will also quantify the predominant themes in order to explore the extent informants concur on areas of concern they identify and to examine any possible associations between these difficulties and clinical symptoms. By adopting a mixed methods analysis we aim to highlight the difficulties young people with TS routinely face in secondary school identified by themselves, their parents and school staff, and so inform strategies to better support them in the future.

## Methods

### Participants

Thirty-five young people with TS, aged between 11 and 18 years, were recruited through the UK charity Tourettes Action (*n* =16) or through secondary schools in the East Midlands, West Midlands and Yorkshire (*n* = 19). Thirty-two were attending mainstream secondary school (Years 7 to 11, Key Stages 3 to 4) and three were in mainstream school sixth form (Years 12 to 13, Key Stage 5). Written confirmation of clinical diagnosis of TS and related co-morbid diagnoses was obtained from participants’ clinicians (written confirmation was not made available for seven of the young people).

All participants gave their informed consent to be interviewed. Parent(s)/carer(s) provided informed consent for their child to participate and also agreed to take part in interviews themselves. In most cases (*n* = 31) the young person’s mother took part in the interviews (a father was interviewed in one case and both parents were present in three cases). Parents and the young people nominated one or two members of school staff to be interviewed. Fifty-four members of school staff were interviewed including Special Educational Needs Coordinators (SENCOs), teaching assistants and teachers.

### Interviews

The young people, parents and staff took part in individual semi-structured interviews about the young person’s current experience of secondary school. Each interview was guided by a schedule of open ended questions such as “Tell me about the ways having Tourette syndrome affects you (your child/this student) in school” and more direct follow-up questions such as “How do you feel Tourette syndrome affects your (his/her) a) class work, b) behaviour in school, c) relationship with other students?”

The interviews took place at the participants’ home or in a private room at school and were carried out by the first author (a researcher trained in qualitative methods) and an experienced Research Nurse. Both had received training in interview techniques. The interviews lasted for between 11 and 53 min and the mean length was 27 min. It is important to note that the interview schedule was designed to encourage participants to talk about how they thought TS affected life in school rather than asking whether participants had, or did not have, a list of specific difficulties. The interview questions were formulated following preliminary qualitative interviews with adolescents with TS and in consultation with parents, clinicians and school staff through stakeholder meetings. The topics explored in the interview schedule were also informed by previous findings of young people with TS experiencing academic, behavioural and social problems (e.g., [17, 19, 22]). The interview audio-recordings were transcribed verbatim and anonymized. Ethical approval was given by The University of Nottingham Medical School Ethics Committee.

### Measures

The following clinical measures were used to assess the young people’s tic severity, OCD, ADHD and ASD symptoms. These measures were administered by the first author who had received training in their use, and were administered after the interview.

- The Yale Global Tic Severity Scale [23] measures the severity of motor and vocal tics. A total tic severity score is given by summing the ratings of type, frequency, duration, intensity, and complexity of motor and vocal tics. Severity scores are classified as mild (1 to 19), moderate (20 to 39) or severe (40 or more). Good psychometric properties have been reported for the use of this measure with children and adolescents [24].
- The Children’s Yale-Brown Obsessive-Compulsive Scale [25] is a 10-item measure of obsessions and compulsions. Ratings of time spent, interference, distress, resistance against and degree of control with regard to obsessions and compulsions are summed to provide an OCD symptom severity score. Scores are classified as subclinical (0 to 7), mild (8 to 15), moderate (16 to 23), severe (24 to 31) and extreme (32 to 40). The authors report good inter-rater reliability, and convergent and discriminant validity have been found to be satisfactory [26].
- ADHD symptom severity was assessed using the Conners 3 Parent-rated Global Index [27]. Scores of 40 to 59 are regarded as average, 60 to 64 as high average, 65 to 60 as elevated and 70 or more as very elevated. The authors report high internal consistency and test–retest validity, and good discriminative and construct validity.
- The Social Communication Questionnaire [28] is a parent-rated screening of ASD symptomatology. A score of 15 or more indicates the possible presence of ASD. The SCQ has been shown to have good discriminant validity [29].

The participants also completed a measure of general cognitive ability, the two sub-test form of the Wechsler Abbreviated Scale of Intelligence [30]. Schools provided information about the young people’s current English and Mathematics level in relation to National Curriculum targets (based on most recent formal teacher assessments at the appropriate Key Stage).

### Analysis approach

The interview transcripts were analysed using theme analysis [31] with the aim of identifying TS-related difficulties in school reported by the participants. Theme analysis allows the researcher to identify themes inductively which appear grounded in the data and deductively based on past research and theory. A coding framework was developed by reviewing literature relevant to TS and educational/school problems. In addition to the empirical articles cited in the introduction, book chapters and articles written by expert clinicians were also reviewed [32–35]. Thus themes represented specific school difficulties (e.g., difficulties in examinations, being teased) and were described in the codebook with examples of exclusions to each theme (with positive and negative examples). Themes were extracted both deductively using the coding framework and inductively when novel TS-related school difficulties were identified. Themes were first coded in the young people’s transcripts. Parents’ and school staff members’ transcripts were then explored for the presence of similar themes and any novel themes not identified in the young people’s interviews. The themes were then organised into superordinate themes.

Next, the most prevalent themes (i.e., school difficulties) from the young people’s accounts were quantified. The difficulties identified by at least 25 % of the young people were tabulated (Table 2) and frequencies and percentages were calculated in relation to the number of young people, parents and staff reporting each school difficulty. The presence or absence of each school difficulty in the participants’ transcripts was coded as 1 (young person reported to have this difficulty) or 0 (young person *not* reported to have this difficulty). In some cases two staff reported for one young person and the school difficulties were coded as present if *either* staff member reported it – thus each school difficulty theme had a maximum frequency of 35 for self-, parent- and staff-report. The level of agreement between the self-, parent- and staff-reports was assessed using Cohen’s Kappa. In order to examine the association between the prevalent school difficulties and severity of motor tics, phonic tics and comorbid clinical symptoms (ADHD, OCD and ASD), the point biserial correlations between the school difficulties and clinical symptom severity were calculated.

## Results and discussion

The clinical characteristics of the participating young people are given in Table 1. There were 33 males and 2 females with a mean age of 13;11 (range 11;4 to 18;5, years; months). The participants’ tic severity scores ranged from mild to severe. Nineteen participants had a diagnosis of TS only and 16 had comorbid conditions (TS+); six had a diagnosis of ADHD and ten had one or more other condition(s) including OCD, ASD, anxiety disorder, attachment disorder, dyslexia and dyspraxia. The group’s mean IQ score was in the expected range (*M* = 95.50, *SD* = 13.87). Most participants were working at or above their Key Stage level, however nine participants (26 %) had not achieved the required level for English and eight (24 %) had not achieved the required level for Maths. Thus, around a quarter of young people were working below national expectations of achievement.

**Table 1:** Participant characteristics

ID	Male or female?	Age (years)	Yale global tic severity^a^	OCD symptom severity^b^	ADHD global index^c^	SCQ lifetime score^d^	ADHD diagnosis?	OCD diagnosis?
01	Female	17	22	16	58	2	No	No
02	Male	13	18	0	-	11	No	No
03	Male	12	32	25	90	16	No	No
04	Male	13	36	9	-	-	No	No
05	Male	12	33	4	76	12	No	No
06	Male	11	38	26	90	11	No	No
07	Male	18	17	7	75	4	No	No
08	Male	14	27	13	90	14	Yes	No
09	Male	12	29	5	80	17	No	Yes
10	Male	15	43	15	90	30	Yes	No
11	Male	12	12	1	90	13	Yes	No
12	Male	14	30	6	90	14	Yes	No
13	Male	11	42	22	55	8	No	No
14	Male	15	45	8	58	10	No	No
15	Male	15	17	12	90	8	No	No
16	Male	12	43	18	90	33	Yes	No
17	Male	13	29	0	72	8	No	No
18	Male	15	17	0	90	13	Yes	No
19	Male	12	37	20	78	21	No	No
20	Male	12	31	19	73	16	No	Yes
21	Male	13	19	7	54	5	No	No
22	Male	14	28	0	65	6	No	No
23	Male	15	39	29	-	-	No	No
24	Male	12	35	16	90	19	Yes	No
25	Male	12	34	16	-	-	Yes	No
26	Male	12	32	24	90	20	Yes	No
27	Male	14	43	0	86	4	Yes	No
28	Male	12	28	6	90	10	Yes	No
29	Male	16	19	4	44	2	No	No
30	Male	13	37	0	68	8	No	No
31	Male	11	28	7	87	10	No	No
32	Male	14	31	20	79	6	No	No
33	Female	12	11	8	-	-	No	No
34	Male	16	17	18	51	5	No	No
35	Male	16	39	26	90	2	No	Yes
M (SD)		13.43 (1.80)	29.66 (9.59)	11.63 (8.99)	77.63 (14.56)	11.87 (7.43)		

^a^Yale Global Tic Severity Scale

^b^Children’s Yale-Brown Obsessive-Compulsive Scale

^c^Conners 3-parent report

^d^Social Communication Questionnaire

All the young people reported difficulties at school related to TS. The majority of the themes were deductive, having been identified in previous academic and clinical TS literature. Three super-ordinate themes were identified from the young people’s interviews, each with a number of subthemes: 1) TS makes school work more difficult, 2) Negative response to TS from staff and fellow students and 3) TS makes it more difficult to manage emotions in school. The themes from the young people’s reports are described below with quotes given to illustrate the subthemes. The themes are then also considered in the context of the school difficulties reported by parents and staff.

### Theme: TS makes school work more difficult

Young people reported that TS can make school work more difficult for them in several ways. TS can cause difficulties with concentration, writing and reading, and completing examinations and homework. The difficulty most commonly described by the young people was concentrating in lessons. The young people talked about finding it difficult to pay attention and finding themselves easily distracted. Some said that the tics themselves were distracting: *“the tics sort of put me off… and I can’t look at the board for long, ‘cause I keep getting tic and I can’t look at it”* (ID 06, 11 years old). Others said trying to suppress tics interfered with concentration: *“when the tics are really bad it does [affect classwork] because I can’t really concentrate on the work, I have to concentrate on keeping the tics, you know, controlling them”* (ID 17, 13 years old)*.* These concentration problems affected the young people’s ability to do their classwork, especially their ability to listen to what the teacher was saying.

Problems with writing were also reported by young people, for example hand tics (e.g., tensing up hands and throwing pens) interfered with their writing. A small number of young people had tics that involved crossing out words and writing them again, or rewriting over the top of words:

*…there was one tic I had about where I had to keep writing over my words over and over again, and I remember one time it just it [the pen] went through the paper and it looked messy and I had to start again and it and it – it was awful my hand was shaking and it was ‘cause it was that painful* (ID 07, 18 years old)

Tics could also interfere with reading, for example, *“I don’t like the fact that when I am reading I will twitch my eyes [eye tic] and I will lose my place and I will have to scan it over again”* (ID 22, 14 years old).

Examinations (both nationally recognized qualifications such as GCSEs and optional teacher assessments) were reported to be challenging by the young people. Feelings of stress and anxiety during the examination could exacerbate tics: *“it’s really nerve-wracking the exam is so, when you’re doing the exam you need, you get more stressed and then they [tics] get worse* (ID 01, 17 years old)*.* Some young people worried about having vocal tics during examinations which would disturb themselves and other students. The young people also said that they found it difficult to complete their homework because of tics and this was a source of stress. *“I just couldn’t control them [tics] to do the homework, so then it was hard. Every night I was spending the whole night doing homework just to get through it”* (ID 01, 17 years old).

The parents also reported that their child had difficulties with concentration, writing and reading, examinations and homework. Some parents described how these concentration difficulties were exacerbated by the classroom environment, for example if it was noisy, with their child finding it easier to concentrate in a more controlled classroom environment:

*But he has terrible trouble when the class isn’t calm and he can’t concentrate. Cos if the other kids are saying things or messing about he is very tempted to say something, to repeat what they have said, and it makes him anxious and his tics are worse. (ID 21, parent)*

A number of parents were aware that in addition to being distracted by others in the classroom, their child’s tics could distract other students in lessons: “*he gets distracted and he distracts others which can be very off-putting for the teacher and that’s the feedback [from school]”* (ID 32, parent)*.* Homework was characterised by parents as a struggle because their child’s tics were worse at home after school and/or due to tiredness after a day in school: “*he does his homework but again he is very tired. I do feel sorry for him when he gets a lot of homework because he has to come home and start again when really he is absolutely worn out” (ID 21, parent).*

Staff also reported TS-related difficulties with school work, particularly problems concentrating. Staff described students as being in their *“own little world”* (ID 24, Form Tutor and teacher) and were concerned that students were not able to engage fully in the content of their lessons. In addition, many staff also noted that students had difficulties in organizing and completing their school work, and needed support in lessons: *“[He] couldn’t function properly in the classroom without [support] because he wouldn’t even finish the task, he probably wouldn’t even start the task”* (ID 24, SENCO). Only a few staff members reported that their student had problems with writing. They described handwriting as “not neat” or “messy” but with no reference to interference specifically from tics. Feelings of anxiety regarding examinations were noted by staff and schools provided special arrangements for some students in examinations, including allowing extra time and/or providing a separate room to work in. There was also an awareness among staff that having tics in school can be very tiring for the student, *“sometimes he just looks at you tired out - it must be quite a physical effort trying to control the tics”* (ID 30, Form Tutor), which can also have an impact on school work.

A number of difficulties with school work were highlighted by young people, parents and staff, most notably concentration problems. TS-related school difficulties and lack of understanding and support can lead to school avoidance/refusal: *“Not knowing how to deal with his specific needs has led to a complete breakdown in him attending full time school”* (ID 03, Learning Support Leader). Strategies such as preferential seating and extended time on classwork and examinations are reported by parents to be particularly helpful strategies [16]. A recent US survey found that using computers for work and assigning an appropriate amount of homework were strategies endorsed by children, parents and teachers [36].

### Theme: Negative response to TS from staff and fellow students

Most young people detailed instances where they felt the response of a staff member following a tic(s) was not helpful. They had, for example, been told off by their teachers for making noises or pulling faces: *“it was in [lesson name] and I was shouting out my word and he told me to shut up twice, and I kept saying ‘Sir I’ve got Tourette’s and that’ – [he] wouldn’t believe me”* (ID 05, 12 years old). A small number had been sent out of the classroom as a result of their tics or disciplined for making inappropriate or offensive comments (coprolalia). These encounters with staff could have significant negative impact on school experience, particularly young people’s willingness to engage in school: *“when I am in a lesson and they have said stuff [about tics], it doesn’t make me want to try as hard, because it feels like they have kind of insulted me”* (ID 30, 13 years old)*.*

Parents also reported that their child experienced unhelpful responses to tics from staff. Being told not to tic was not a helpful strategy in the classroom, according to parents. Parents encountered unhelpful attitudes from staff in meetings and telephone conversations, for example, *“His tutor told us that there was nothing wrong with him, he was just a naughty boy”* (ID 04, parent). In most cases, these unhelpful responses were restricted to only a few individuals in the school, and the participants did also identify staff members who had been very supportive and managed the students’ tics and associated symptoms well. Unsurprisingly, relatively few staff members reported that receiving unhelpful comments and/or responses from other staff was a problem for the student. Those that did emphasised the need to educate other staff members about TS and to remind them about the student’s profile and how to support them. *“So it’s changing the mindset really, from X being naughty to X having this real problem”* (ID 30, Form Tutor). The need for better understanding of TS in schools is indicated by the number of reports of school staff responding inappropriately to tics. Encouraging teachers to ignore tics has been reported to be a helpful strategy [16]. European clinical guidelines highlight the need to improve understanding of the TS in schools [14] and there is some evidence that teacher workshops can lead to a small but significant increase in knowledge of TS [37].

The majority of the young people reported that other students in the school had reacted negatively towards them because of their TS. Other students stared at them and told them to “stop it” or “shut up”. They also described being laughed at and being made fun of and some peers imitated their tics.

*Usually the people that copy or imitate my tics, that’s the worst people can do. They don’t realise how it can make me feel. There was this one boy once who came up to me once who said to me my worst tics and kept saying them* (ID 27, 14 years old)

Having difficulties with other students in school was also reported by many parents, and other students mimicking tics was notably problematic. There was also a sense that continual “low level” teasing was having a detrimental effect on their child: “*It’s not a case of one major bully, it’s five or six minor little digs for the individual but if you get that from five or six different people in one class in one day, for X it’s huge”* (ID 27, parent). This type of teasing behaviour may be hard for schools to tackle; *“[the teasing] is endless and school deals with one group of children that are doing it and then there is another groups of kids that are doing it, it is really quite bad”* (ID 28 parent). Some parents reported specific incidents where their child had been physically victimized by peers in school, *“children in his year at school, a lot of them still try and bully him and push him and I think somebody shut the door on his hand”* (ID 19, parent).

In contrast, relatively few staff reported that their student had difficulties with other students such as teasing and name-calling. This behaviour may occur outside the classroom during times of minimal staff supervision (e.g., lunch time and on the school bus) and students may not report these incidents to staff. Peer education has been shown to improve the knowledge and attitudes of peers and classmates towards an individual with TS [38, 39] and this may be a valuable strategy to use in schools if a student is at risk of teasing or bullying.

A number of parents and staff members were concerned that the young person had no friends and was socially isolated in school (inductive theme): *“It [TS] has had huge knock on effect on his friendship groups and peer groups which is what we are finding most hurtful, he has become very isolated”* (ID 15, parent)*.* In some cases this social isolation was the result of negative experiences with peers such as bullying and teasing, for example, *“he is becoming more and more isolated from peers because peers just do not know how to handle [him]”* (ID 26, SENCO). In a Swedish study, 66 % of children with TS were rated by teachers as having social problems such as having no friends and problems with empathy and social understanding [10]. Thus, some students with TS may need socioemotional support in school.

### Theme: TS makes it more difficult to manage emotions in school

Feelings of stress and anxiety in school were reported by young people*.* Sources of stress included concerns about letting tics out in school, getting school work completed and examinations. Young people also reported that they easily became angry at school and/or had a quick temper. In some cases this had resulted in aggressive behaviour towards staff and students. Young people thought that these problems with anger were directly associated with having TS.

*I’m trying to stop doing them [tics] and it builds up tension and I start to get angry and angry and I let it out on people, and that can be quite annoying ‘cause you know it’s not really their fault, it’s something else like inside of me* (ID 07, 18 years old)*.*

Some parents also reported their child had problems managing anger in school which had resulted in incidents of physical aggression against self and others, threatening behaviour and damage to property. In describing an incident involving a teacher, a parent said: *“I think the teacher was quite worried I think, over his temper and he starts crying when he’s that angry. You know when he gets like that… he goes from OK to completely head butting walls”* (ID 12, parent). A larger number of parents reported anxiety issues in school. Specific sources of anxiety reported included worries about not being able to complete class work and being in large crowds. The relationship between anxiety in school and tic exacerbation was highlighted by a number of parents:

*When he gets stressed [by class work] the tics and noises become more sort of pronounced and he then gets told off by the teachers…so he then gets even more stressed and then it just progresses onto more tics which he doesn’t want to have, so he is stressed about that as well, so it is just a vicious circle really* (ID 16, parent)*.*

Many of the staff interviewed reported that their student appeared to be anxious or stressed in school. Some staff felt that the young person was anxious about what behaviors or tics they might do and whether other people will notice. *“he’s a little bit anxious I think about the fact that his tics are quite pronounced and he’s a bit worried about his classmates I think noticing that”* (ID 08, teacher). Fewer staff reported anger management to be an issue for their student in school.

### Prevalent school difficulties

The most prevalent school difficulties, reported by over 25 % of young people with TS, are given in Table 2 along with the frequencies from parent- and staff-reports. The eight difficulties most frequently reported by young people included problems with school work (concentration, homework, examinations and writing), difficulties with staff and other students, and emotional issues (anxiety and managing anger in school). These school difficulties were also reported by over 25 % of the parents interviewed. More than 25 % of staff reported concentration and anxiety problems, but the other difficulties were reported less frequently by staff.

**Table 2:** Difficulties in secondary school reported by young people with TS, parents and staff

Type of school difficulty	Self-report frequency (percentage)	Parent-report frequency (percentage)	Staff-report frequency (percentage)
Concentration	22/35 (62.9 %)	23/35 (65.7 %)	18/35 (51.4 %)
Unhelpful staff response to tics	20/35 (57.1 %)	21/35 (60.0 %)	7/35 (20.0 %)
Difficulties with other students	20/35 (57.1 %)	16/35 (45.7 %)	7/35 (20.0 %)
Homework	15/35 (42.8 %)	18/35 (51.4 %)	8/35 (22.9 %)
Examinations	15/35 (42.8 %)	11/35 (31.4 %)	7/35 (20.0 %)
Anxiety in school	10/35 (28.6 %)	12/35 (34.3 %)	17/35 (48.6 %)
Writing	9/35 (25.7 %)	10/35 (28.6 %)	5/35 (14.3 %)
Managing anger in school	9/35 (25.7 %)	9/35 (25.7 %)	6/35 (17.1 %)

The most prevalent TS-related difficulty in secondary school was concentration in line with previous US survey findings [15]. This was clearly identified as a barrier to learning by the participants. Homework was especially problematic from the perspective of parents but fewer staff reported the student had homework difficulties. School staff may be unaware that a student struggles to complete homework, because he/she tics more at home and/or are tired from suppressing tics during the school day.

Many young people and parents reported incidents where school staff had not responded well to tics, which can be upsetting for young people and frustrating for parents to deal with. However, far fewer members of school staff reported this to be a challenge for their student. This is not surprising as individual members of staff are unlikely to be aware of specific incidents occurring throughout the school day, although staff may also have been unwilling to criticize colleagues. As noted by the staff interviewed and in a recent survey of school staff, increasing knowledge of TS among staff is an important strategy in supporting students with TS and could address these issues to some extent [40].

TS can also affect young people with TS socially in secondary school and previous US studies report that around a quarter of young people with TS experience bullying [17, 18]. In this study, more than half of the young people reported some degree of teasing or negative comments from peers in school, but the staff were much less aware of these issues.

Although staff reported fewer TS-related class work and social difficulties, almost half of school staff reported that their student was anxious in school. The relationship between feelings of stress/anxiety and tic exacerbation is well documented [41], and a number of staff and parents had observed a link between anxiety levels and tic severity in the young person at school. Controlling feelings of anger in school was also a challenge highlighted by participants. Young people with TS are at risk of anger problems and around 40 % of children with TS are reported to have recurrent episodes of explosive anger or aggression, which are regarded as one of the more disabling aspects of TS [42, 43]. Anger issues in school were reported by around a quarter of the young people, and the parent reports suggest incidents involving anger outbursts can have serious consequences.

The self-, parent- and staff-reports agreed as to the type of TS-related difficulties most frequently experienced by the young people in secondary school, but these problems tended to be reported less frequently by staff. For some of these difficulties, staff members’ main source of information may be the child and/or parent rather than direct experience (e.g., staff may not be present when teasing occurs).

### Agreement between self-, parent- and staff-reports

Table 3 gives the degree of agreement between informants regarding the presence/absence of the most prevalent school difficulties for the 35 young people. Agreement between self- and parent-reports ranged from slight to substantial [44]. There was good agreement regarding difficulties with other students and writing difficulties, and modest agreement regarding unhelpful staff responses and anxiety in school. Agreement levels between self- and staff-reports of these school difficulties was less good (ranging from slight to modest agreement), but there was a modest agreement regarding individuals having homework problems and unhelpful staff responses. Agreement levels between parent- and staff-reports also ranged from slight to moderate agreement. Parents and staff demonstrated moderate agreement regarding problems with homework, examinations and writing. Thus, the extent to which the three informants agreed on the specific difficulties faced by the young person varied considerably. Self-, parent- and staff-reports did not strongly agree as to which difficulties individual students had in secondary school.

**Table 3:** Agreement between self-, parent- and staff-reports of school difficulties

School difficulties	Agreement between self- and parent-report	Agreement between self- and staff-report	Agreement between parent- and staff-report
	Kappa [95 % CI]	Kappa [95 % CI]	Kappa [95 % CI]
Concentration	.07 [−.27, .40]	.08 [−.24, .40]	.14 [−.18, .45]
Unhelpful staff response to tics	.35 [.04, .67]*	.32 [.01, .53]*	.18 [−.03, .40]
Difficulties with other students	.66 [.42, .90]**	.00 [−.24, .24]	.10 [−.18, .38]
Homework	.26 [−.06, .58]	.32 [.03, 61]*	.44 [.19, .68]**
Examinations	.16 [−.17, .48]	.13 [−.17, .42]	.41 [.09, .75]*
Anxiety in school	.34 [.01, .67]*	.02 [−.29, .32]	.14 [−.18, .45]
Writing	.64 [.35, .93]**	.30 [−.06, .66]	.42 [.09, .76]*
Managing anger in school	.10 [−.24, .45]	.08 [−.26, .42]	.25 [−.12,.61]

Note. Kappa < 0 = no agreement, 0 to 0.20 = slight agreement, 0.21 to 0.40 = fair agreement, 0.41 to 0.60 = moderate agreement, 0.61 to 0.80 = substantial agreement, 0.81 to 1.00 = almost perfect agreement [44]

*p < .05. **p < .01

Young people and parents tended to be in better agreement with each other than with staff, particularly with regard to difficulties with peers. Parents and staff agreed more on difficulties related to academic work (homework, examinations and writing). Interestingly, although concentration problems were reported by over half of all informants, the level of agreement as to which individual students had difficulties concentrating was poor. A previous study comparing parent- and teacher-reported child difficulties found agreement that concentration and learning difficulties were present but parents were more concerned about these problems and rated them as more severe [45]. The authors concluded that lack of agreement between parent and teacher reports is a source of potential conflict between home and school.

### Associations between self-reported school difficulties and clinical symptoms

Point biserial correlations were calculated between tic severity (motor and phonic) and the eight most frequently school difficulties reported by the young people. Motor tic severity was significantly and positively correlated with homework problems (*r*_*pb*_ = .39, *p* = .02) and with writing problems (*r*_*pb*_ = .44, *p* = .01). Phonic tic severity was significantly and positively correlated with reporting unhelpful staff responses (*r*_*pb*_ = .46, *p* = .01). ADHD, OCD and ASD symptom severity were not significantly correlated with any of the eight prevalent school difficulties. The relative lack of associations between school difficulties and specific clinical symptoms may reflect the multi-faceted nature of TS-related school problems but the relatively small sample size should also be taken into account.

### Implications

TS can affect a young person’s ability to do school work, their interactions with others and coping with emotions they experience in school. One aim of this study was to give young people with TS a voice with respect to their school experience, which can be valuable both in research and in developing support strategies [46]. This study highlights the need to ask young people with TS about what they find hard and what would help them in school. Staff tended to report fewer and different TS-related school difficulties compared to parents and young people. Thus, effective communication between families and schools may be valuable in identifying problem areas and appropriate support. Only three (prevalent) school difficulties were found to be significantly correlated with tic severity. This suggests that the severity of a student’s tics alone is not necessarily a good indication of the degree of interference from TS they can experience in school, or indeed the level of support needed.

### Limitations

The use of semi-structured interviews to explore school difficulties provided rich data but did not allow measurement of the regularity, severity and level of impairment associated with these problems. The findings of the study relate to a self-/parent-selected sample of young people who were attending mainstream secondary school, and many were already receiving support via school and/or the TS support organisation. Nonetheless, a range of TS-related difficulties in secondary school were reported and some were very prevalent. Just under half of the young people interviewed had additional diagnosis (e.g., ADHD) that may also contribute to problems in school. Although participants were asked to focus on TS-related difficulties, we acknowledge that many of these difficulties may also be associated with co-morbid conditions.

The school staff members who took part in the research were selected by the participating families and were likely to be individuals they had repeated contact with or who supported the young person in school. As such, the staff members who were interviewed probably had more experience and understanding of TS than other staff members within their schools. Future research could seek to examine the views of teachers and other school staff with less experience of TS.

## Conclusions

Concentration difficulties, unhelpful responses from staff and difficulties with other students emerged as the most common TS related concerns for students. More problems with staff responses were associated with greater tic severity but generally concerns were not related to level of symptoms. There was some recognition from staff that students may experience unhelpful responses from teachers and other school staff and limited recognition of students’ perceptions of difficulties with peers. Anxiety emerged as a key issue identified in half the interviews with staff and over a quarter of interviews with students. The impact of anxiety on management of TS in school emerged as an overarching theme.

Given the difficulties reported in relation to school staff and other students it is clear that understanding and empathy should be promoted in schools, by educating staff and students about TS. By examining the most frequently reported school difficulties, this study suggests that those supporting young people with TS should be particularly aware of 1) concentration difficulties, 2) unhelpful responses to tics from staff and 3) teasing from other students, whilst also being mindful that TS can present challenges in many different ways in the school environment.
